# Low Systemic Inflammation Response Index Predicts Good Prognosis in Locally Advanced Pancreatic Carcinoma Patients Treated with Concurrent Chemoradiotherapy

**DOI:** 10.1155/2020/5701949

**Published:** 2020-07-30

**Authors:** Erkan Topkan, Huseyin Mertsoylu, Ahmet Kucuk, Ali Ayberk Besen, Ahmet Sezer, Duygu Sezen, Yasemin Bolukbasi, Ugur Selek, Berrin Pehlivan

**Affiliations:** ^1^Department of Radiation Oncology, Baskent University Medical Faculty, Adana, Turkey; ^2^Department of Medical Oncology, Baskent University Medical Faculty, Adana, Turkey; ^3^Mersin City Education and Research Hospital, Radiation Oncology Clinics, Mersin, Turkey; ^4^Department of Radiation Oncology, Koc University School of Medicine, Istanbul, Turkey; ^5^Department of Radiation Oncology, The University of Texas, MD Anderson Cancer Center, Houston, TX, USA; ^6^Department of Radiation Oncology, Bahcesehir University, Istanbul/, Turkey

## Abstract

**Background:**

We investigated the prognostic significance of pretreatment systemic inflammation response index (SIRI) in locally advanced pancreatic carcinoma (LAPC) patients treated with concurrent chemoradiotherapy (CRT).

**Methods:**

Present retrospective cohort analysis investigated consecutive 154 LAPC patients who received radical CRT. The SIRI was defined as: SIRI = neutrophil × monocyte/lymphocyte counts. Ideal SIRI cutoff(s) influencing overall survival (OS) and progression-free survival (PFS) results were sought by using receiver operating characteristic (ROC) curve analysis. The primary endpoint was the interaction between the SIRI and OS results.

**Results:**

The median follow-up, PFS, and OS durations were 14.3 (range: 2.9-74.6), 7.9 [%95 confidence interval (CI): 5.7-10.1), and 14.7 months (%95 CI: 11.4-18.0) for the entire cohort, respectively. ROC curve analyses determined the ideal SIRI cutoff that exhibiting a significant link with OS and PFS outcomes at the rounded 1.6 point (AUC: 74.3%; sensitivity: 73.8%; specificity: 70.1%).The SIRI <1.6 patients (*N* = 58) had significantly superior median PFS (13.8 versus 6.7 months; *P* < 0.001) and OS (28.6 versus 12.6 months; *P* < 0.001) lengths than SIRI ≥1.6 patients (*N* = 96), respectively. Although the N0 (versus N1; *P* < 0.05) and CA 19-9 ≤90 U/mL (versus >90 U/mL) appeared as the other significant associates of better OS and PFS in univariate analyses, yet the results of multivariate analyses confirmed the SIRI <1.6 as the independent indicator of superior OS and PFS (*P* < 0.001 for each).

**Conclusion:**

Pretreatment SIRI is a novel independent prognosticator that may further enhance the conventional tumor-node-metastases staging system in a more precise prediction of the OS and PFS outcomes of LAPC patients after radical CRT.

## 1. Introduction

Approximately, 30% of all pancreatic cancer (PC) patients present with nonmetastatic but locally advanced unresectable disease, namely locally advanced PC (LAPC) [1]. The prognosis of such patients is gloomy with an estimated survival ranging between 8 and 12 months [2]. Although the current care standard for LAPCs is highly debated, yet, chemoradiotherapy (CRT) remains as a widely recognized viable treatment option for such patients due to the postmortem observations assigning 30% of all PC-related deaths to locoregionally uncontrolled LAPCs, rather than a widespread metastatic disease status which is quite frequent in such patients [3, 4].

Cancer-related local and/or systemic inflammation has been spotted as the seventh hallmark of cancer [5]. Inflammation is not only an independent risk factor for initiation, progression, and dissemination ventures of PC, but it may equally emerge as a tumor-associated reactionary process, as well. In this regard, multiple epidemiological studies have assuredly confirmed the inflammation as a triggering component of PC development and progression [6]. Additionally, a burgeoning amount of literature suggests systemic inflammation as one of the principal factors determining the distinctive patient prognoses following identical treatment regimens [7–11]. The blood-born circulating indicators of the systemic inflammation typically include the neutrophils, monocytes, platelets, lymphocytes, and albumin and C-reactive protein. These key indicators have been broadly investigated and shown to dependably predict the prognosis of PC patients after various treatment modalities [12–15].

Recently, Qi et al. [16] have shown that the systemic inflammation response index [SIRI = neutrophil (N) × monocyte (M)/lymphocyte (L)] was able to reliably laminate PC patients into two unique prognostic groups after chemotherapy with regards to the time to progression (TTP) and overall survival (OS). Likewise, in a succeeding study, SIRI was found to be also connected with clinical outcomes of metastatic PC patients who received FOLFIRINOX (fluorouracil, leucovorin, irinotecan, and oxaliplatin) chemotherapy [17]. Further studies in renal clear cell [18], hepatocellular [19], nasopharyngeal [20], and esophageal squamous cell carcinomas [21] affirmed the prognostic worthiness of SIRI in these tumor types, as well. Nevertheless, the value of SIRI in the prediction of clinical outcomes of LAPC has never been addressed in the context of LAPC patients undergoing exclusive CRT. Therefore, the present retrospective cohort analysis was carried out to investigate the prognostic efficiency of SIRI in newly diagnosed LAPC patients managed with definitive CRT.

## 2. Patients and Methods

### 2.1. Study Population

The institutional database of Baskent University Medical Faculty Department of Radiation Oncology was retrospectively sought to identify all technically unresectable LAPCs treated with radical CRT between January 2007 and December 2017. To be unresectable, primary tumors had to involve the celiac axis and/or superior mesenteric artery, namely stage III (T4N0-1M0) disease according to the AJCC staging framework (7^th^ ed.). Our institutional standards for diagnostic and staging workup for such patients were as previously reported elsewhere [22, 23]. In brief, all eligible patients underwent abdominal magnetic resonance imaging (MRI), magnetic resonance cholangiopancreatography (MRCP), and endoscopic ultrasonography (if underwent open abdominal exploration) for abdominal disease staging; diagnostic thoracic computed tomography (CT) for thoracic involvement, and brain MRI for exclusion of brain metastasis. Additionally, all patients underwent ^18^F-fluorodeoxyglucose (FDG)-positron emission tomography (PET)-CT to better detect the probable distant metastases. Patients had to meet the following additional requirements to be eligible for this review analysis: age 18 to 80 years, Eastern Cooperative Oncology Group (ECOG) performance status 0-1, proven adenocarcinoma histology, absent prior chemotherapy/RT history, body mass index (BMI) >20 kg/m^2^, and adequate pre-CRT routine complete blood counts and biochemistry tests, to be assessed for conversion surgery after the completion of the CRT. The research protocol was approved by the Institutional Ethical Committee before any data acquisition, and the investigation was performed under the Helsinki Declaration and Rules of Good Clinical Practice.

### 2.2. Concurrent Chemoradiotherapy

As detailed previously [22, 23], all patients received radical CRT comprising a dose of 45 Gy RT (1.8 Gy/fraction, 5 days/week, for 5 weeks) which exclusively encompassed the primary tumor site and involved nodes with no allowance to elective nodal irradiation per our institutional norms for such patients. All patients received continuously infused 5-fluorouracil (225 mg/m^2^/day) over the span of RT that was trailed by 2 to 6 courses of maintenance gemcitabine (1000 mg/m^2^ intravenously on days 1 and 8 every 21 days).

### 2.3. Measures of Systemic Inflammation Response Index

As per the Qi and colleagues' original definition, the baseline SIRI was calculated for each patient by utilizing the total blood count tests obtained on the first day of CRT [16]: SIRI = neutrophil (N) × monocyte (M)/lymphocyte (L).

### 2.4. Treatment Response Evaluation

Following the completion of CRT, the treatment response was evaluated every 3 and 6 monthly intervals for the first 2 years and >2 years of follow-up, respectively. Restaging PET/CT and abdominal MRI/CT scans were utilized for this goal and reported responses indicated the best result according to the objective criteria defined by the EORTC 1999 guidelines. PET/CT was replaced by abdominal MRI/CT scans in cases with a confirmed complete metabolic response at any time point during the follow-up period. Additionally, total blood count and biochemistry tests, serum CA 19-9 concentrations were obtained at each visit. Any of the abdominal ultrasonography, chest CT, cranial MRI, and/or bone scintigraphy examinations were accomplished merely if disease progression was suspected.

### 2.5. Statistical Analysis

Our primary endpoint was to examine the difference between the overall survival (OS: the interval between the first day of CRT and the date of death/last follow-up) outcomes of the patients with high versus low pretreatment SIRI measures, while our secondary endpoint was the progression-free survival (PFS: the interval between the first day of CRT and the dates of any type of disease progression/death/last follow-up) per SIRI group. Frequency distributions were used for categorical variables, while medians and ranges were utilized to describe continuous variables. Student's *t*-tests, chi-square tests, or Spearman's correlation analyses were used to compare the correlations between patients groups, as needed. Accessibility of pre-CRT SIRI cutoff(s) that may group the study population into two SIRI gatherings with distinctive OS and PFS results was searched for by performing receiver operating characteristic (ROC) curve analysis. The potential link between different covariates and OS and PFS were analyzed with Kaplan-Meier estimates and log-rank tests. The multivariate Cox proportional hazard model was utilized to assess potential interactions between these variables and survival outcomes. Any two-sided *P* value <0.05 was considered significant.

## 3. Results

A total of 154 patients sufficing the eligibility criteria of the present research protocol were identified. Baseline patient and disease characteristics were as portrayed in Table 1. The median age was 57 years (range: 29-79) for the entire study cohort. Of all 154 patients, 120 (77.9%) were males. Pancreatic head (*n* = 124; 80.5%) involvement was more common than the body/tail (*n* = 30; 19.5%) regions, and 73 (47.4%) cases harbored N1 disease.

During a median follow-up period of 14.3 months (range: 2.9-74.6), 48 (31.2%) patients were still alive with respective 47 (31.2%) and 25 (16.2%) locoregional recurrence- and progression-free rates. Widespread systemic metastases were the evident cause of death in 103 (96.3%) of all 107 deaths. The median and 4-year survival rates were 14.7 months [%95 confidence interval (CI): 11.4-18.0] and 21.4% for OS and 7.9 months (95% CI: 5.7-10.1) and 11.7% for PFS, respectively. All patients were evaluated for conversion surgery 6 weeks after the completion of surgery. Only 17 (11.0%) patients were deemed to be suitable for conversion surgery, with 14 achieving a R0 resection.

**Figure 1:**
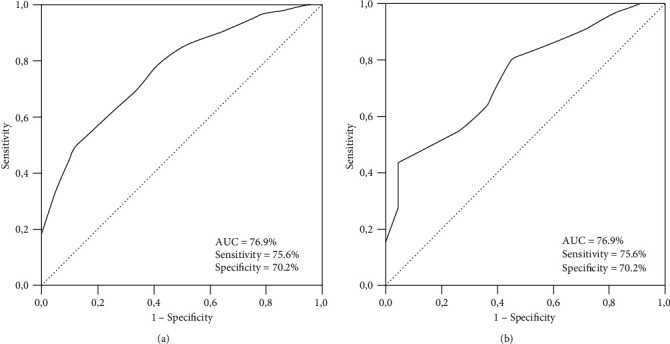
Receiver operating curve analyses outcomes: (a) progression-free survival, and (b) overall survival.

We performed ROC curve analysis to assess the accessibility of the optimal cutoff(s) for pre-CRT SIRI values linking significantly with OS and PFS results. The optimal SIRI cutoffs were determined at 1.62 [area under the curve (AUC): 76.9%; sensitivity: 75.4%; specificity: 70.2%] and 1.59 (AUC: 74.3%; sensitivity: 73.8%; specificity: 70.1%) values for PFS and OS, respectively (Figure 1). Because the two cutoffs were remarkably close, we used the rounded 1.6 as the optimal cutoff for patients grouping and further intergroup comparisons: group 1: SIRI<1.6 (*n* = 58), and group 2: SIRI≥1.6 (*n* = 96), respectively.

**Figure 2:**
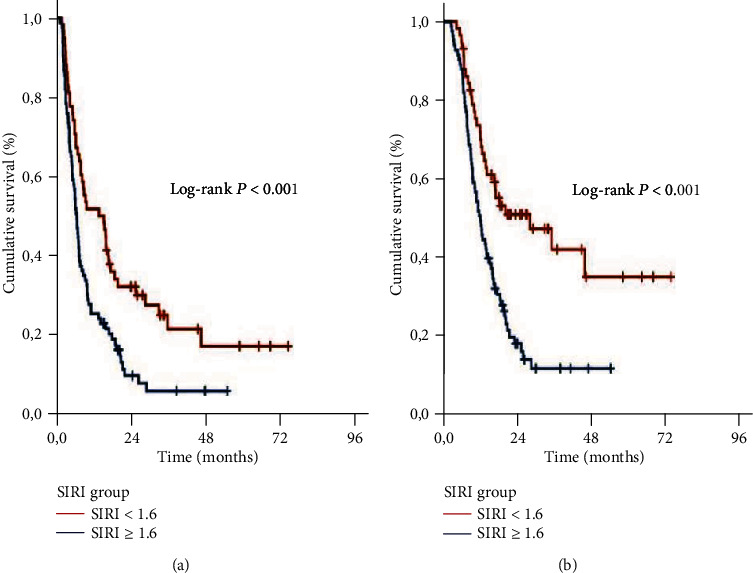
Survival outcomes per systemic inflammation response index (SIRI) groups: (a) progression-free survival, and (b) overall survival.

The Kaplan-Meier curves per SIRI group attested that the SIRI <1.6 patients had significantly longer median PFS (15.9 versus 6.0 months; *P* < 0.001) and OS (25.8 versus 11.4 months; *P* < 0.001) durations than their SIRI ≥1.6 companion. Besides, asserting the persistency of the favorable prognosis at longer lengths, the 2- and 4-year PFS and OS rates were likewise numerically superior in the SIRI <1.6 than the SIRI ≥1.6 group (Figure 2). Considering the suitability for conversion surgery, we could not demonstrate any meaningful difference between the two SIRI groups (13.8% for SIRI <1.6 versus 9.6% for SIRI ≥1.6; *P* = 0.018).

Factors depicted in Table 1 were tested in univariate analyses for the significance of their influence on the OS and PFS outcomes (Table 2), which revealed the N0 stage (versus N1), CA 19-9 ≤90 U/mL (versus >90 U/mL), and SIRI <1.6 (versus ≥1.6) as the favorable factors to predict better survival outcomes for each endpoint (*P* < 0.05 for each). The CA 19-9 cutoff was set at 90 U/mL which was utilized as the critical cutoff in the landmark Charité Onkologie 001 (CONKO-001) randomized trial [24]. As portrayed in Table 2, the results of multivariate analyses incorporating only these factors confirmed that all three factors had separate independent prognostic significance on PFS (*P* < 0.05, for each) and OS (*P* < 0.05, for each) results. Accordingly, the patients in the N0 stage, CA 19-9 ≤90 U/mL, and SIRI <1.6 had significantly superior 2- and 4-year survival lengths than their respective N1 stage, CA 19-9 >90 U/mL, and SIRI ≥1.6 counterparts (Table 3).

## 4. Discussion

To our best information, the present study represents the first attempt to assess the prognostic significance of the baseline SIRI in LAPC patients treated with radical CRT. The SIRI <1.6 was discovered to be connected with significantly superior PFS and OS results, which suggested an independent and serviceable prognostic worth for SIRI in further separation of such patients into two discrete prognostic subgroups surpassing the conventional TNM staging framework.

Indeed, the complex relationship between the inflammation and carcinogenesis, tumor growth, and metastasis dates back to Virchow's unique proposition in the 19^th^ century [25], which has been proven almost more than a century and established as one of the hallmarks of cancer [26–28]. A gradually growing body of proof uncovered that the circulating neutrophils, monocytes, macrophages, lymphocytes, platelets, acute-phase reactants albumin, and C-reactive protein, and cancer-related chemokines and cytokines were involved in the local and systemic inflammation during the carcinogenesis and progression phases of numerous cancers, including the LAPC [27–29]. Since the systemic inflammation has been shown to diminish the host immunity, facilitate escape from immune surveillance, inhibit apoptosis and promote neoangiogenesis, tumor aggressiveness, invasion capacity, and metastasis; various indexes of systemic inflammation response including the NLR, PLR, MLR, PNI, PI, Glasgow prognostic score (GPS), modified GPS, and ALI have been examined broadly in patients presenting with various cancers and demonstrated to reliably predict their prognosis [7–15]. The SIRI, another recently discovered indicator of systemic inflammation, has been shown to be a novel relevant index that can accurately predict the prognosis of patients with pancreatic [16, 17], renal clear cell [18], hepatocellular [19], nasopharyngeal [20], and esophageal squamous cell carcinomas [21] who received various anticancer treatments. Nevertheless, despite its first discovery in advanced PC patients, it was surprising for us to notice that the SIRI was never examined before in LAPC patients who experienced radical CRT. Thus, principally encouraged with the previously mentioned fundamental information, we conducted this current retrospective analysis so as to explore the potential prognostic utility of the baseline SIRI in LAPC patients receiving radical CRT.

The best fit treatment for unresectable LAPC patients has not been established yet, with radical CRT, induction chemotherapy followed by RT or CRT, systemic chemotherapy alone, and upfront stereotactic body radiotherapy (SBRT) in select cases being the viable treatment options. Considering the most commonly used induction chemotherapy followed by CRT and systemic chemotherapy alone, the survival outcomes of our present study appears to be in good concordance with the reported outcomes of such studies. In a 2016 meta-analysis of 11 LAPC studies comprising 315 patients with available PFS and OS results, Suker et al. [30] reported that the median pooled PFS and OS from the start of FOLFIRINOX regimen was 10.0 months (95% CI: 21.7-26.8) and 15.0 months (95% CI: 13.8-16.2), respectively, which are better than our 7.9 and 14.7 months. However, this discrepancy seems to be associated with the inclusion of borderline resectable and unresectable LAPCs in the same analysis in Suker's meta-analysis: borderline resectable patients constituted one-fourth to one-third of all patients. Although the survival benefit of CRT after induction chemotherapy is limited [31], yet, it appears to improve local control rates [32].

Although our results confirmed the prognostic values of well-recognized N-stage and CA19-9 status, yet, the essential novel finding of our current investigation was the show of vigorous prognostic estimation of pretreatment SIRI on the OS (*P* < 0.001) and PFS (*P* < 0.001) of LAPC patients who underwent radical CRT. It is quite difficult for us to comment conclusively on this finding in the lack of similarly designed comparative past LAPC studies. In any case, our display SIRI cutoff esteem at 1.6 and the independent link between the prevalent survival outcomes in patients with SIRI <1.6 appear to be in good accordance with the results of historic chemotherapy studies investigating the prognostic worth of SIRI in PC patients [16, 17]. In the first of ever SIRI study, Qi et al. [16] tested the SIRI in advanced PCs receiving palliative chemotherapy and showed that the SIRI <1.8 group had notably longer median TTP (*P* = 0.003) and OS (*P* < 0.001) durations than the SIRI ≥1.8 group. In view of these results, the authors counseled the use of SIRI as a novel predictor of survival for advanced PC patients receiving chemotherapy and proposed its use for the identification of the appropriate candidates for aggressive therapy in the routine oncology practice. These findings were later asserted in a subsequent SIRI study in metastatic PC patients who received FOLFIRINOX chemotherapy with an ideal cutoff defined at 1.9 points [17]. Proposing an almost universal prognostic utility for SIRI in discrimination of patients groups with distinctive outcomes, further investigations verified these findings in the renal clear cell, hepatocellular, nasopharyngeal, and esophageal squamous cell carcinomas [18–21], as well.

The precise mechanism(s) underlying the causal relationship between low SIRI values and superior clinical results in LAPC patients has not been ascertained yet. However, since the immune and inflammatory cells comprise nearly 50% of LAPC tumor load [33], some sound remarks can be made by considering the original SIRI formula: SIRI = neutrophils × monocytes/lymphocytes. Lymphocytes are the main determinant of the host immune response against the malignant cells; hence, inflammation-related any decrease in lymphocyte counts reflects a critically depressed immune surveillance and host defense mechanisms against cancer cells, while normal or increased lymphocyte counts conversely indicate an intact immune response [34]. Resultantly, high lymphocyte counts will indicate a lower SIRI and better clinical results. On the other hand, neutrophils and monocytes are involved in the stimulation of inflammatory chemokines and cytokines, promotion of cancer cell proliferation, invasiveness, metastatic potential, and induction of resistance to anticancer treatments [35]. Additionally, the derivatives of monocytes, to be specific the tumor-associated macrophages, may further promote the abovementioned unfavorable functions of neutrophils. Any increase in neutrophils and monocytes will consequently reflect higher tumor loads and aggravated inflammatory status [36]. Therefore, a low SIRI reflects a highly active systemic immunity prompted by lymphocytes against the cancer cells which outperform and refute the proinflammatory and cancer cell promoting functions of neutrophils and monocytes. This favorable immune-inflammation status may sensibly explain the superior outcomes observed in cancer patients presenting with low SIRI levels. Moreover, the SII, another comprehensive immune-inflammation index, has been recently shown that SII >900 [hazard ratio (HR): 2.32] was associated with significantly diminished cancer-specific survival an associate of 590 PC patients by Aziz et al. [37]. Considering the fact that only the monocytes of SIRI are replaced by the platelets in the SII formula, taken together, Aziz et al. study and the one introduced here altogether propose critical roles for complex immune-inflammation response indices in the lamination of PC patients to further prognostic groups past that of the traditional staging system.

Our present investigation is strengthened with the restrictive inclusion of LAPC patients who experienced a thorough staging procedure; including contrast-enhanced abdominal CT, MRI, MRCP, PET-CT, laparotomy/laparoscopy, and histopathologic exclusion of suspected metastasis in any metabolically active lesion. Taken collectively with the use of a single CRT protocol in all cases, these properties confirm the relative homogeneity of our study population, which enhances the relevancy of the results introduced here. On the other side, our study likewise has some specific impediments. First, our results represent the outcomes of a single institutional retrospective cohort analysis in a relatively small cohort size which might be biased by the various unforeseeable host or tumor-related confounders. Therefore, they must only be respected as hypothesis-generating until the publication of further confirmatory studies on this critically important issue. Second, there is a risk for selection bias as we deliberately included only the patients who underwent CRT with an excellent performance status (ECOG 0-1), which does not reflect the whole real-world practices in such patients. Third, the uncontrollable possible practice differences among the referring centers during the adjuvant and or salvage treatment phases may have altered the outcomes in favor of one group. Fourth, we restricted our investigation to the pre-CRT measures. Nevertheless, because the SIRI is a dynamic biomarker, its measures may fluctuate during the CRT and follow-up periods in strong relation with the changes in the tumor load, host immune response, and systemic inflammation status. Therefore, the dynamic changes of SIRI may potentially mirror the response to treatment or conversely the tumor progression earlier than the apparent radiographic changes. Accordingly, forthcoming well-designed studies should focus on the presence of a nadir SIRI cutoff which may reliably predict the prognosis of LAPCs undergoing CRT. And fifth, notwithstanding the fact that either of the systemic chemotherapy alone, induction chemotherapy trailed by CRT, or upfront SBRT separately represents the other viable treatment options for such patients, our study design did not address the most suited treatment per SIRI levels. This was mainly due to the selection of patients who were decided to undergo CRT by our institutional gastrointestinal tumors board. In any case, still, because the 11.4 months OS of SIRI ≥1.6 cohort observed here was essentially similar to the survival durations reported for metastatic PCs [38], it is prudent to contemplate that chemotherapy alone or induction chemotherapy trailed by chemoradiotherapy, or upfront SBRT may represent wiser treatment choices in selected cases, which may spare such patients from the futile complications of the unnecessary chemoradiotherapy.

## 5. Conclusion

The consistent results of the present investigation showed that the pretreatment SIRI was an independent prognostic factor for LAPC patients undergoing radical CRT which may enhance the prognostic utility of the conventional TNM staging system. If confirmed with further large-scale studies, this novel finding may serve profitably in the guidance of customized treatments for such patients.

## Figures and Tables

**Table 1:** Baseline patient and disease characteristics.

Characteristics	All patients (*N* = 154)	SIRI <1.6 (*N* = 58)	SIRI ≥1.6 (*N* = 96)	*P* value
Median age, years (range)	57 (29-79)	55 (31-79)	58 (29-78)	0.86
Age group (*N*; %)				
<70 years	121 (78.6)	44 (75.9)	77 (80.2)	0.21
≥70 years	33 (21.4)	14 (24.1)	19 (19.8)	
Gender (*N*; %)				
Female	34 (22.1)	13 (23.4)	21 (21.9)	0.85
Male	120 (77.9)	45 (77.6)	75 (78.1)	
ECOG performance (*N*; %)				
0	62 (40.2)	22 (37.9)	40 (41.7)	0.69
1	92 (59.8)	36 (62.1)	56 (58.3)	
Tumor location (*N*; %)				
Head	124 (80.5)	47 (81.0)	77 (80.2)	0.92
Body/tail	30 (19.5)	11 (19.0)	19 (19.8)	
N-stage (*N*; %)				
0	81 (52.6)	30 (51.7)	51 (53.1)	0.77
1	73 (47.4)	28 (48.3)	45 (46.9)	
CA 19-9 (*N*; %)				
≤90 U/mL	48 (31.2)	21 (36.2)	27 (28.1)	0.18
>90 U/mL	106 (68.8)	37 (63.8)	69 (71.9)	
Median neutrophils (10^3^/mm^3^)	4.21	3.53	5.84	0.001
Median M/L	0.38	0.34	0.44	0.002

Abbreviations: ALI: advanced lung cancer inflammation index; ECOG: Eastern Cooperative Oncology Group; N-stage: nodal stage; CA 19-9: cancer antigen 19-9; BMI: body mass index; M/L: monocyte/lymphocyte.

**Table 2:** Outcomes of uni- and multivariate analysis.

Factor	OS			PFS		
	Univariate *P* value	Multivariate *P* value	HR	Univariate *P* value	Multivariate *P* value	HR
Age group (<70 vs. ≥70 y)	0.74	—	—	0.69	—	—
Gender (female vs. male)	0.78	—	—	0.83	—	—
ECOG (0 vs. 1)	0.89	—	—	0.76	—	—
Tumor location (H vs. B/T)	0.76	—	—	0.84	—	—
N-stage (0 vs. 1)	0.006	0.011	2.49	0.012	0.018	2.66
CA 19-9 (< vs. ≥ 90 U/m/L)	0.014	0.019	1.53	0.008	0.015	2.10
SIRI (< vs. ≥1.6)	<0.001	<0.001	2.97	<0.001	<0.001	3.31

Abbreviations: OS: overall survival; PFS: progression-free survival; HR: hazard ratio; ECOG: Eastern Cooperative Oncology Group; H: head; B/H: body/tail; N-stage: nodal stage; CA 19-9: cancer antigen 19-9; SIRI: systemic inflammation response index.

**Table 3:** Survival outcomes according to the factors exhibiting independent prognostic value in multivariate analyses.

Survival	N_0_ (*N* = 69)	N_1_ (*N* = 85)	*P* value	CA 19-9 ≤90 U/m/L (*N* = 55)	CA 19-9 >90 U/mL (*N* = 99)	*P* value	SIRI <1.6 (*N* = 58)	SIRI ≥1.6 (*N* = 96)	*P* value
OS									
Median, mo	21.4	10.6	0.011	20.3	13.7	0.016	28.6	12.6	<0.001
2-year (%)	43.9	14.2		44.8	27.4		51.0	18.2	
4-year (%)	26.6	10.7		28.5	18.6		35.1	11.8	
PFS									
Median, mo	12.4	6.3	0.018	10.2	5.8	0.022	13.8	6.7	<0.001
2-year (%)	27.2	7.8		29.4	14.6		32.1	9.7	
4-year (%)	15.3	5.7		16.2	7.7		17.1	5.8	

Abbreviations: N_0/1_: nodal stage 0/1; CA 19-9: cancer antigen 19-9; SIRI: systemic inflammation response index; OS: overall survival; PFS: progression-free survival.

## Data Availability

Data cannot be shared publicly because the data is owned and saved by Baskent University Medical Faculty. Data are available from the Baskent University Radiation Oncology Institutional Data Access/Ethics Committee (contact via Baskent University Ethics Committee) for researchers who meet the criteria for access to confidential data: contact address, adanabaskent@baskent.edu.tr.

## References

[1] Yeo T. P., Hruban R. H., Leach S. D. (2002). Pancreatic cancer. *Current Problems in Cancer*.

[2] Johung K., Saif M. W., Chang B. W. (2012). Treatment of locally advanced pancreatic cancer: the role of radiation therapy. *International Journal of Radiation Oncology • Biology • Physics*.

[3] Huguet F., Girard N., Guerche C. S.-E., Hennequin C., Mornex F., Azria D. (2009). Chemoradiotherapy in the management of locally advanced pancreatic carcinoma: a qualitative systematic review. *Journal of Clinical Oncology*.

[4] Iacobuzio-Donahue C. A., Fu B., Yachida S. (2009). DPC4 gene status of the primary carcinoma correlates with patterns of failure in patients with pancreatic cancer. *Journal of Clinical Oncology*.

[5] Hanahan D., Weinberg R. A. (2011). Hallmarks of cancer: the next generation. *Cell*.

[6] Raimondi S., Lowenfels A. B., Morselli-Labate A. M., Maisonneuve P., Pezzilli R. (2010). Pancreatic cancer in chronic pancreatitis; aetiology, incidence, and early detection. *Best Practice & Research. Clinical Gastroenterology*.

[7] Martin H. L., Ohara K., Kiberu A., van Hagen T., Davidson A., Khattak M. A. (2014). Prognostic value of systemic inflammation-based markers in advanced pancreatic cancer. *Internal Medicine Journal*.

[8] Birtolo C., Go V. L. W., Ptasznik A., Eibl G., Pandol S. J. (2016). Phosphatidylinositol 3-Kinase. *Pancreas*.

[9] Steele C. W., Jamieson N. B., Evans T. R. J. (2013). Exploiting inflammation for therapeutic gain in pancreatic cancer. *British Journal of Cancer*.

[10] Zambirinis C. P., Pushalkar S., Saxena D., Miller G. (2014). Pancreatic cancer, inflammation, and microbiome. *Cancer Journal*.

[11] McMillan D. C. (2013). The systemic inflammation-based Glasgow Prognostic Score: a decade of experience in patients with cancer. *Cancer Treatment Reviews*.

[12] Shimizu T., Taniguchi K., Asakuma M. (2019). Lymphocyte-to-monocyte ratio and prognostic nutritional index predict poor prognosis in patients on chemotherapy for unresectable pancreatic cancer. *Anticancer Research*.

[13] Xiao Y., Xie Z., Shao Z. (2017). Prognostic value of postdiagnostic inflammation-based scores in short-term overall survival of advanced pancreatic ductal adenocarcinoma patients. *Medicine*.

[14] Shirai Y., Shiba H., Haruki K. (2017). Preoperative platelet-to-albumin ratio predicts prognosis of patients with pancreatic ductal adenocarcinoma after pancreatic resection. *Anticancer Research*.

[15] Yamada S., Fujii T., Yabusaki N. (2016). Clinical implication of inflammation-based prognostic score in pancreatic cancer: Glasgow Prognostic Score is the most reliable parameter. *Medicine*.

[16] Qi Q., Zhuang L., Shen Y. (2016). A novel systemic inflammation response index (SIRI) for predicting the survival of patients with pancreatic cancer after chemotherapy. *Cancer*.

[17] Pacheco-Barcia V., Mondéjar Solís R., France T. (2019). A systemic inflammation response index could be a predictive factor for mFOLFIRINOX in metastatic pancreatic cancer. *Pancreas*.

[18] Chen Z., Wang K., Lu H. (2019). Systemic inflammation response index predicts prognosis in patients with clear cell renal cell carcinoma: a propensity score-matched analysis. *Cancer Management and Research*.

[19] Xu L., Yu S., Zhuang L. (2017). Systemic inflammation response index (SIRI) predicts prognosis in hepatocellular carcinoma patients. *Oncotarget*.

[20] Chen Y., Jiang W., Xi D. (2019). Development and validation of nomogram based on SIRI for predicting the clinical outcome in patients with nasopharyngeal carcinomas. *Journal of Investigative Medicine*.

[21] Geng Y., Zhu D., Wu C. (2018). A novel systemic inflammation response index (SIRI) for predicting postoperative survival of patients with esophageal squamous cell carcinoma. *International Immunopharmacology*.

[22] Topkan E., Yavuz A. A., Aydin M., Onal C., Yapar F., Yavuz M. N. (2008). Comparison of CT and PET-CT based planning of radiation therapy in locally advanced pancreatic carcinoma. *Journal of Experimental & Clinical Cancer Research*.

[23] Yildirim B. A., Ozdemir Y., Colakoglu T., Topkan E. (2016). Impact of presence and degree of pretreatment weight loss in locally-advanced pancreatic cancer patients treated with definitive concurrent chemoradiotherapy. *Pancreatology*.

[24] Oettle H., Post S., Neuhaus P. (2007). Adjuvant chemotherapy with gemcitabine vs observation in patients undergoing curative-intent resection of pancreatic cancer: a randomized controlled trial. *Journal of the American Medical Association*.

[25] Balkwill F., Mantovani A. (2001). Inflammation and cancer: back to Virchow?. *The Lancet*.

[26] Germano G., Allavena P., Mantovani A. (2008). Cytokines as a key component of cancer-related inflammation. *Cytokine*.

[27] Mantovani A., Allavena P., Sica A., Balkwill F. (2008). Cancer-related inflammation. *Nature*.

[28] Coussens L. M., Werb Z. (2002). Inflammation and cancer. *Nature*.

[29] Elinav E., Nowarski R., Thaiss C. A., Hu B., Jin C., Flavell R. A. (2013). Inflammation-induced cancer: crosstalk between tumours, immune cells and microorganisms. *Nature Reviews. Cancer*.

[30] Suker M., Beumer B. R., Sadot E. (2016). FOLFIRINOX for locally advanced pancreatic cancer: a systematic review and patient-level meta-analysis. *The Lancet Oncology*.

[31] Wang C., Liu X., Wang X., Wang Y., Cha N. (2018). Effects of chemoradiotherapy and chemotherapy on survival of patients with locally advanced pancreatic cancer: a meta-analysis of randomized controlled trials. *Medicine*.

[32] Mukherjee S., Hurt C. N., Bridgewater J. (2013). Gemcitabine-based or capecitabine-based chemoradiotherapy for locally advanced pancreatic cancer (SCALOP): a multicentre, randomised, phase 2 trial. *The Lancet Oncology*.

[33] Balkwill F. R., Capasso M., Hagemann T. (2013). The tumor microenvironment at a glance. *Journal of Cell Science*.

[34] Ferrone C., Dranoff G. (2010). Dual roles for immunity in gastrointestinal cancers. *Journal of Clinical Oncology*.

[35] Diakos C. I., Charles K. A., McMillan D. C., Clarke S. J. (2014). Cancer-related inflammation and treatment effectiveness. *The Lancet Oncology*.

[36] Shibutani M., Maeda K., Nagahara H. (2015). Prognostic significance of the lymphocyte-to-monocyte ratio in patients with metastatic colorectal cancer. *World Journal of Gastroenterology*.

[37] Aziz M. H., Sideras K., Aziz N. A. (2019). The systemic-immune-inflammation index independently predicts survival and recurrence in resectable pancreatic cancer and its prognostic value depends on bilirubin levels: A retrospective multicenter cohort study. *Annals of Surgery*.

[38] Azar I., Virk G., Esfandiarifard S., Wazir A., Mehdi S. (2019). Treatment and survival rates of stage IV pancreatic cancer at VA hospitals: a nation-wide study. *Journal of Gastrointestinal Oncology*.

